# Targeting CD157 in AML using a novel, Fc-engineered antibody construct

**DOI:** 10.18632/oncotarget.16060

**Published:** 2017-03-09

**Authors:** Christina Krupka, Felix S. Lichtenegger, Thomas Köhnke, Jan Bögeholz, Veit Bücklein, Michael Roiss, Torben Altmann, To Uyen Do, Rachel Dusek, Keith Wilson, Arnima Bisht, Jon Terrett, Dee Aud, Esteban Pombo-Villar, Christian Rohlff, Wolfgang Hiddemann, Marion Subklewe

**Affiliations:** ^1^ Department of Internal Medicine III, Klinikum of The LMU Munich, Munich, Germany; ^2^ Clinical Cooperation Group Immunotherapy at The Helmholtz Institute Munich, Munich, Germany; ^3^ Laboratory of Translational Cancer Immunology, Gene Center Munich, Ludwig-Maximilians-University, Munich, Germany; ^4^ Department of Hematology, University Hospital Zurich, Zurich, Switzerland; ^5^ Independent consultant Oxford BioTherapeutics Ltd, Abingdon, United Kingdom and San Jose, CA, USA; ^6^ CRISPR Therapeutics, Cambridge, MA, USA

**Keywords:** AML, Immunotherapy, CD157, Antibody, Fc-engineering

## Abstract

Antibody-based immunotherapy represents a promising strategy to eliminate chemorefractory leukemic cells in acute myeloid leukemia (AML). In this study, we evaluated a novel Fc-engineered antibody against CD157 (MEN1112) for its suitability as immunotherapy in AML. CD157 was expressed in 97% of primary AML patient samples. A significant, albeit lower expression level of CD157 was observed within the compartment of leukemia-initiating cells, which are supposed to be the major source of relapse. In healthy donor bone marrow, CD157 was expressed on CD34^+^ cells. In *ex vivo* assays, MEN1112 triggered natural killer (NK) cell-mediated cytotoxicity against AML cell lines and primary AML cells. Compared to its parental analogue, the Fc-engineered antibody exhibited higher antibody dependent cellular cytotoxicity responses. Using NK cells from AML patients, we observed heterogeneous MEN1112-mediated cytotoxicity against AML cells, most likely due to well-documented defects in AML-NK cells and corresponding inter-patient variations in NK cell function. Cytotoxicity could not be correlated to the time after completion of chemotherapy. In summary, we could demonstrate that CD157 is strongly expressed in AML. MEN1112 is a promising antibody construct that showed high cytotoxicity against AML cells and warrants further clinical testing. Due to variability in NK-cell function of AML patients, the time of application during the course of the disease as well as combinatorial strategies might influence treatment results.

## INTRODUCTION

Overall survival of patients with acute myeloid leukemia (AML) has remained poor, despite decades of clinical studies. This is mainly due to residual leukemic cells, surviving standard chemotherapeutic regimens and inducing high relapse rates of > 50%. There is a high need to develop new treatment options. Immunotherapy has shown remarkable success for various cancer entities in recent years [1, 2], and antibody-based immunotherapeutic strategies are currently evolving quickly.

The primary target antigen in AML has long been CD33. The most prominent anti-CD33 antibody in clinical application is gemtuzumab ozogamicin (GO, Mylotarg, Pfizer, New York, NY, USA), conjugated to calicheamicin. After a changeful history with accelerated approval in 2000 [3] and voluntary withdrawal from the market in 2010 [4], a meta-analysis of five randomized controlled trials showed that the addition of GO to conventional chemotherapy significantly reduced the risk of relapse and resulted in a survival benefit for cytogenetically favorable and intermediate-risk group patients. However, a significant proportion of patients do still not benefit from GO treatment [5]. Apart from variations in linker and conjugation partners, the choice of the target antigen has the potential for further improvement of antibody-based therapy in AML.

Antibodies directed at a multitude of antigens are under development, among others CD123 [6, 7], CLL-1 [8, 9], CD44 [10, 11], and CD47 [12]. Here we report data on a new antibody directed at CD157. This molecule was originally cloned under the name of bone marrow stromal cell antigen 1 (BST-1) and found to facilitate pre-B-cell growth [13]. It is a glycosyl-phosphatidylinositol (GPI)-anchored membrane protein with close resemblance to CD38 [14, 15]. CD157 is detectable on several cell types, such as dermal fibroblasts and peritoneal mesothelial cells, but mainly on myeloid cells in peripheral blood mononuclear cells (PBMCs) [16]. Flow cytometric analysis of normal bone marrow (BM) revealed that CD157 expression becomes positive at the stage of CD34^−/low^ myeloblasts and is high on more mature neutrophils and particularly on monocytic cells [17, 18].

With respect to hematological malignancies, CD157 is significantly higher expressed on B-cell precursor acute lymphoid leukemia (ALL) cells compared to normal B-cell populations in the BM.

In this study, we are the first to report data of CD157 expression on AML samples. CD157 could be validated as a strongly expressed target antigen in AML. Furthermore, we tested the *ex vivo* activity of MEN1112, an Fc-optimized anti-CD157 antibody.

MEN1112 induced efficient lysis of AML cell lines and primary AML cells *ex vivo* in an allogeneic and autologous setting. However, in comparison to healthy NK cells, we observed reduced cytotoxicity using NK cells from AML patients.

Taken together, the results obtained in this study encourage further clinical development of MEN1112.

## RESULTS

### CD157 is frequently expressed in primary AML patient samples

**Figure 1:**
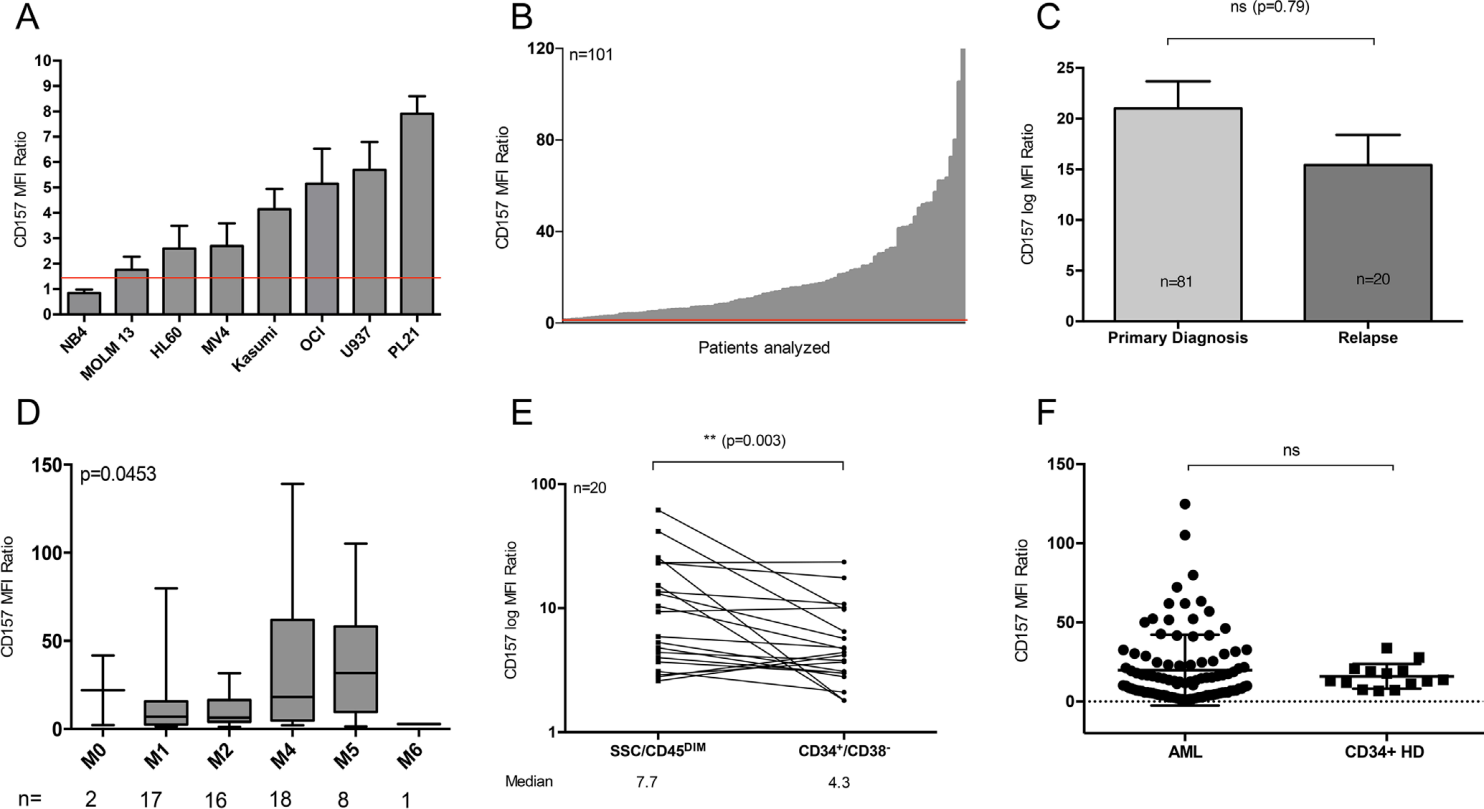
Ubiquitous CD157 expression in AML (A + B) Flow cytometry-based CD157 expression analysis (**A**) on 8 AML cell lines and in (**B**) 101 primary AML patient samples at primary diagnosis or relapse. Median fluorescence intensity (MFI) ratio was determined as a measure of expression intensity (see Materials and Methods). The red line indicates an MFI ratio of 1.5, indicating CD157 positivity. (**C**) Comparison of CD157 expression intensity (MFI ratio) at primary diagnosis (*n =* 81) vs. relapse (*n =* 20; *p =* 0.79). (**D**) CD157 expression intensity correlated to French American British (FAB) subtypes (**E**) CD157 expression intensity (MFI ratio) on CD34^+^/CD38^−^ leukemia initiating cells (LICs) compared to leukemic bulk cells (SSC/CD45^DIM^) (*n =* 20; *p =* 0.003) (**F**) Expression of CD157 on CD34^+^ bulk cells in bone marrow (BM) samples from healthy donors (HDs) (*n =* 14) compared to leukemic bulk cells (SSC/CD45^DIM^) (*n =* 101). **p < 0.01, ****p < 0.0001, ns *p* > 0.5.

We first determined CD157 expression intensity (median fluorescence intensity; MFI ratio) on 8 AML cell lines. 7/8 cell lines were found to express surface CD157 (MOLM-13, HL60, MV4-11, Kasumi-1, OCI-AML3, U937 and PL21). Positive cell lines (MFI ratio > 1.5) showed variable expression intensities of CD157, with PL21 showing the highest (median MFI ratio 8.6, *n* = 3) and MOLM-13 the lowest (median MFI ratio 1.8, *n* = 4) MFI ratio (Figure 1A). The intensity of CD157 expression was further evaluated in 101 samples of newly diagnosed or relapsed AML patients. In 97% (98/101) of samples, positivity for CD157 could be demonstrated with substantial inter-patient heterogeneity in expression levels (Figure 1B). The direct comparison of CD157 and CD33 expression within the same patient cohort revealed lower expression of the former (*n* = 101, median MFI ratio CD33 vs CD157: 59.3 vs 12.5; Supplementary Figure 1). Due to relevant differences in antibody conjugated fluorochromes, statistical analysis was not performed. Comparison of CD157 expression at primary diagnosis and at time of relapse revealed no significant difference in expression intensity (*n* = 81 at primary diagnosis, *n* = 20 at relapse, *p* = 0.79, Figure 1C). To determine any correlation with cytogenetic or molecular disease characteristics, the patient cohort was subdivided into halves based on CD157 MFI ratio (Supplementary Figure 2). High CD157 expression levels correlated with the prognostically adverse group of patients according to the European Leukemia Net (ELN) classification (*p* = 0.03). In contrast, no significant difference in prevalence among halves was determined for *NPM1* and *FLT3-ITD* mutational status (*p* = 0.25) (Supplementary Figure 2). Among the entire patient cohort, CD157 expression was significantly different between FAB-subgroups (*p* = 0.0453) with M4 and M5 subtypes showing the highest mean expression (mean MFI ratio 41.3 and 34.1, respectively) (Figure 1D).

As leukemia-initiating cells (LICs) – most frequently found within the CD34^+^/CD38^−^ cell compartment – are supposed to be the source of relapse, we next analyzed the expression level of CD157 on CD45^DIM^, CD34^+^/CD38^−^ cells of AML patients in comparison to leukemic bulk cells (CD45^DIM^). We found significantly lower CD157 expression on the former (median MFI ratio 4.3, *n* = 20) compared to the latter (median MFI ratio 7.7, *p* = 0.003; Figure 1E)

Comparison of CD34^+^ progenitor cells from HD BM to CD45^DIM^ AML cells revealed no significant difference in CD157 expression level (*p* = 0.4; Figure 1F).

### MEN1112 shows high cytotoxicity against CD157^+^ AML cell lines

**Figure 2:**
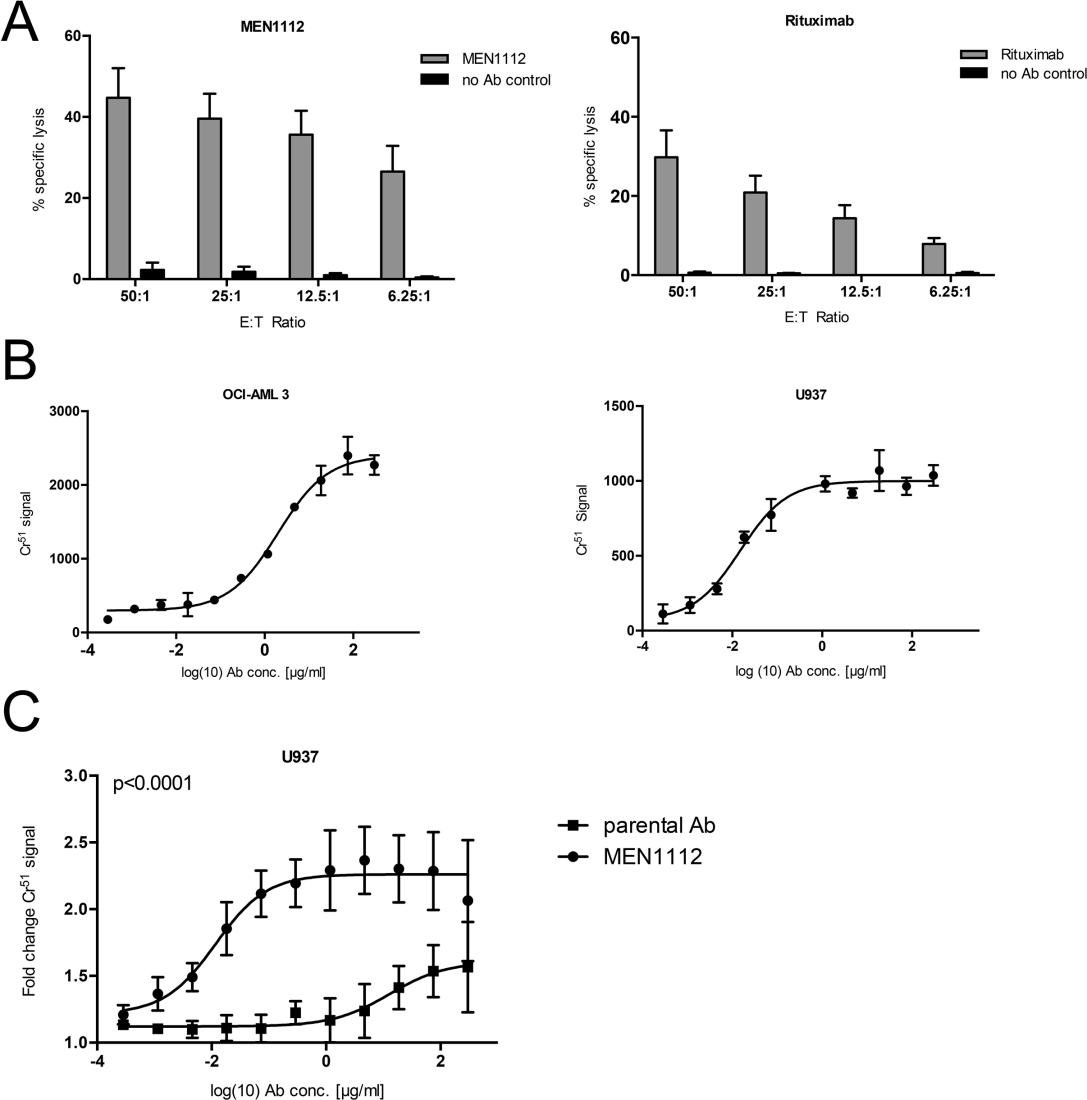
MEN1112 mediates cytotoxicity against AML cell lines (**A**–**C**) Standard 4h-Cr^51^ release cytotoxicity experiments using (A) different concentrations of HD natural killer (NK) cells (E:T ratios 50:1-6.25:1) and either CD157-expressing OCI-AML3 cells and MEN1112 (10 μg/ml; left panel) or CD20-expressing RAJI cells and Rituximab (10 μg/ml; right panel). Data represent mean ± SEM of 3 independent experiments (B) EC_50_ determination of MEN1112 in OCI-AML3 (left panel) and U937 (right panel) cells. The graphs are representative for 1 out of 3 experiments. EC_50_ values for all 3 experiments are listed in Table 1. (C) Cytotoxicity of MEN1112 against U937 cells compared to its parental analogue (parental Ab). Fold change compared to target cells and NK cells without antibody is demonstrated.

MEN1112-mediated cytotoxicity was evaluated in standard 4h Cr^51^ release experiments. Using HD NK cells, MEN1112 mediated cytotoxicity against OCI-AML3 cells compared to control cultures in an effector to target (E:T) ratio-dependent manner (*n* = 3). Compared to MEN1112-triggered lysis of AML cells, non Fc-engineered Rituximab (anti-CD20 antibody) mediated reduced lysis of RAJI cells (CD20^+^ MFI ratio: 12.3), especially at low E:T ratios, despite equal surface expression intensities of their respective target antigens (*n* = 3; Figure 2A). Next, we determined the half maximum lysis capacity (EC_50_) for MEN1112 in dose response titration experiments using OCI-AML3 and U937 as target cells. MEN1112 mediated dose-dependent lysis of OCI-AML3 and U937 cells with a median EC_50_ of 2.35 μg/ml and 0.009 μg/ml, respectively (Figure 2B and Table 1). Furthermore, MEN1112 demonstrated superior cytotoxic activity on U937 cells compared to its parental analogue, which was not enhanced for Fc-receptor binding (*p* < 0.0001; Figure 2C).

**Table 1:** EC_50_ values of ADCC experiments

Experiment #	EC_50_ (μg/ml) OCI-AML3	EC_50_ (μg/ml) U937
1	2.0	0.009
2	2.7	0.005
3	3.0	0.02
4	1.9	n.d.

n.d., not determined, EC_50_, half maximal effective concentration.

### MEN1112 shows a trend towards higher cytotoxicity against AML cells compared to CD34^+^ BM progenitor cells

**Figure 3:**
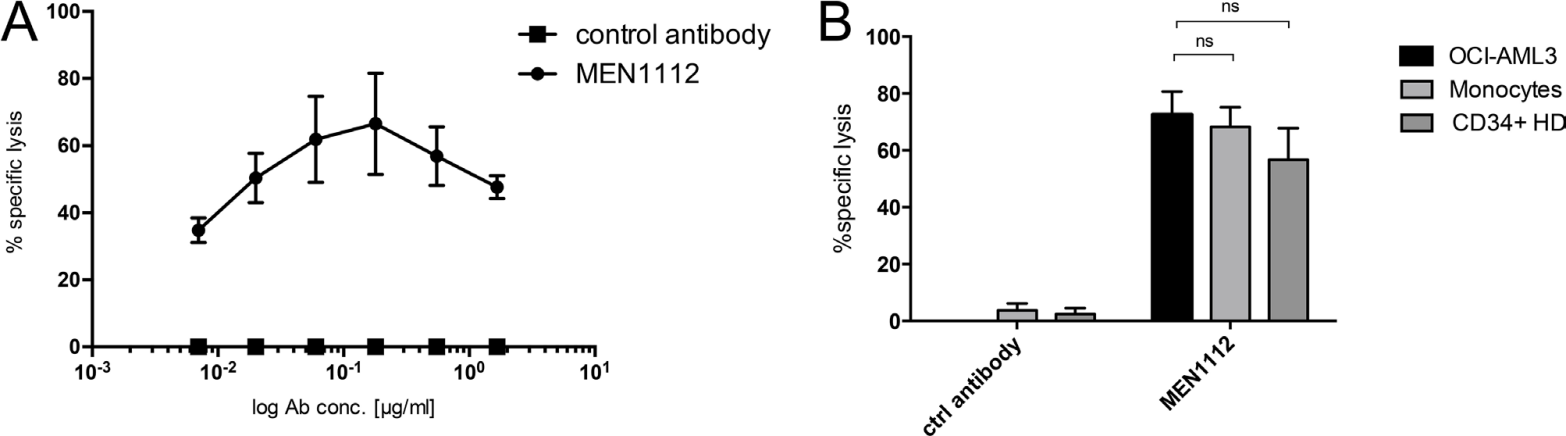
Limited cytotoxicity of MEN1112 on healthy CD34^+^ bone marrow progenitor cells (A + B) Flow cytometry-based cytotoxicity experiments using (**A**) healthy human peripheral blood mononuclear cells (PBMCs) incubated with MEN1112 or an irrelevant Fc-engineered control antibody (control antibody) (1.7-0.007 μg/ml) for 20-24 hours. Depletion of monocytes within the PBMC sample is shown. Data represent mean ± SEM of 2 independent experiments. (**B**) 24 hour-MEN1112 cytotoxicity assay using healthy donor (HD) NK cells and either OCI-AML3 cells, monocytes or CD34^+^ healthy bone marrow (BM) cells at an E:T ratio of 3:1. MEN1112 or the irrelevant Fc-engineered control antibody were added at 5 μg/ml.

As we have shown that CD157 is also expressed on CD34^+^ BM progenitor cells (see Figure 1), we evaluated the cytotoxic effects of MEN1112 on this cell population as well as on monocytes, which were also demonstrated to express CD157 [18]. The addition of MEN1112 to HD PBMCs resulted in dose-dependent elimination of monocytes within the sample (*n* = 2; Figure 3A).

However, the direct comparison of OCI-AML3 cells, monocytes and CD34^+^ BM progenitor cells in coculture with HD NK cells revealed that MEN1112 shows a trend towards higher cytotoxicity against leukemic cells compared to CD157-expressiong healthy CD34^+^ BM progenitor cells (*n* = 6, Figure 3B).

### NK cells from AML patients show impaired antibody-mediated cytotoxicity compared to NK cells from HDs

**Figure 4:**
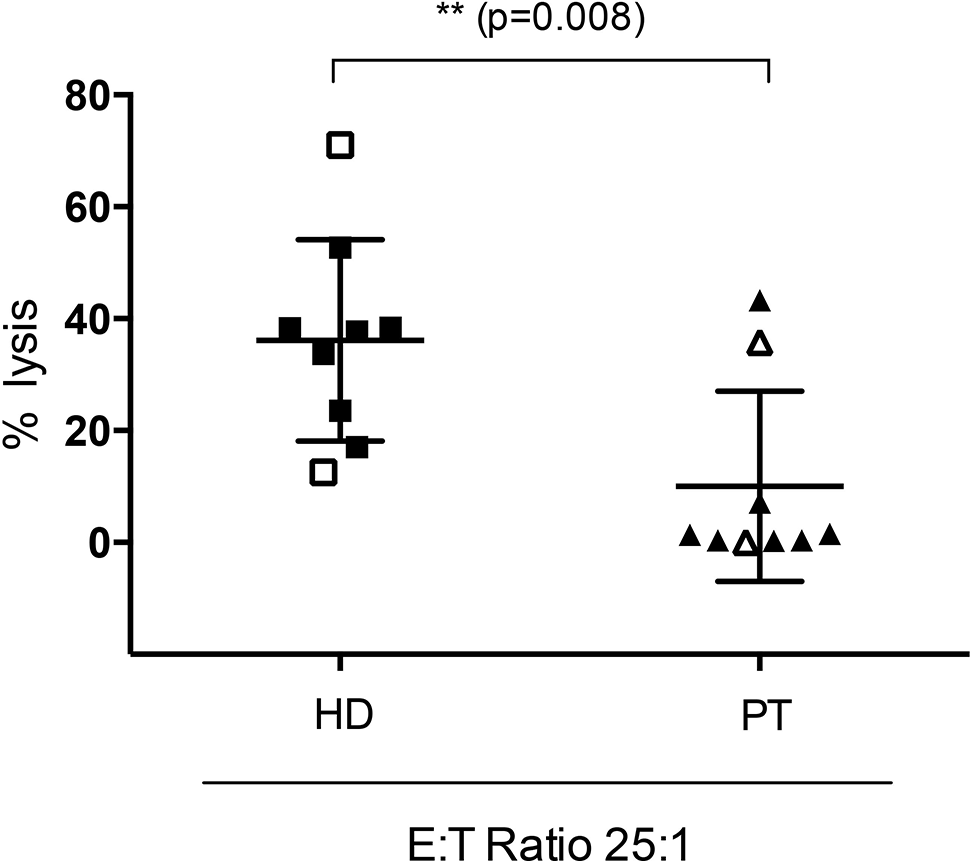
Reduced antibody-mediated cytotoxicity using NK cells from AML patients Standard 4h-Cr^51^ release cytotoxicity experiments using NK cells from healthy donors (HDs) vs AML patients (PT) in complete remission (at different time points within the treatment course) at different E:T ratios (50:1-3.125:1). The AML cell lines OCI-AML3 and U937 (closed squares or triangles) and the lymphoma cell line RAJI (open squares or triangles) were used as target cells. MEN1112 or Rituximab were used at 10 μg/ml. Target cells and NK cells without antibody served as negative control. Lysis efficacy was highly variable between AML patients but could not be correlated to time after completion of chemotherapy. Results from an E:T ratio of 25:1 are depicted in the graph.

We compared the cytotoxic potential of NK cells from HDs to those from AML patients using either the AML cell lines OCI-AML3 or U937 and MEN1112 or the lymphoma cell line RAJI and Rituximab. NK cells were obtained from AML patients at complete remission at different time points after completion of chemotherapy. In 4 h Cr^51^ release cytotoxicity experiments, heterogeneous results could be obtained: In 2/9 samples, cytotoxicity of NK cells from AML patients was comparable to NK cells from HDs. In another sample, MEN1112-mediated lysis could be detected but was significantly reduced compared to NK cells from HDs. In the remaining 6 samples, no lysis of AML cells could be obtained using NK cells from AML patients. Cytotoxicity could not be correlated to time after completion of chemotherapy. Table 2 and Figure 4 summarize patient characteristics and corresponding results from cytotoxicity experiments.

**Table 2:** Patient characteristics

PT	Gender	AML Blasts at Dx (%)	FAB	*NPM1*mut	*FLT3-ITD*	Karyotype	ELN genetic group	Frontline Therapy	NK cell source (treatment phase)	% specific lysis at E:T ratio 25:1
1A	M	56	M2	–	–	aberrant	Intermediate I	sHAM	pre-consolidation (1st cycle) pre-maintenance (1st cycle) post-maintenance (4th cycle)	0.4
1B										35.8
										7.1
1C										
2	W	70	M4	+	–	normal	favorable	TAD/HAM	pre-consolidation (2nd cycle)	0.0
3	M	17	M1	+	–	n.d.	n.d.	AraC+Daunorubicin (7 + 3)	post-consolidation (2nd cycle)	0.3
4	M	60	M4	+	–	normal	favorable	sHAM	pre-consolidation (1st cycle)	43.3
5	M	80	M4	+	–	normal	favorable	AraC+Daunorubicin (7 + 3)	pre-maintanance (12th cycle)	1.4
6	M	67	M3	–	–	normal	Intermediate I	PETHEMA	pre-maintanance (1st cycle)	1.5
7	W	19	sAML	–	–	complex	adverse	sHAM	pre-maintanance (1st cycle)*	0.4
8	M	90	M4	+	+	normal	Intermediate I	n.a.	HD	19.0
9	F	90	M1	+	+	aberrant	Intermediate II	n.a.	HD	17.4
10	F	n.d.	n.d.	n.d.	n.d.	n.d.	n.d.	n.a.	HD	9.5
11	F	72	M1	+	+	normal	Intermediate I	n.a.	HD	7.5
12	F	76	M1	+	–	normal	favorable	n.a.	HD	10.9
13	M	42	M2	–	–	normal	Intermediate I	n.a.	PD	6.9^†^
14	M	40	M2	n.d.	n.d.	n.d.	n.d.	n.a.	PD	33.1^†^
15	F	63	M4eo	–	–	aberrant	favorable	modified sHAM/AraC+Daunorubicin (7 + 3)	post-maintenance (4th cycle)	21.1^†^

^*^ received no consolidation; PT, patient; PD, primary diagnosis; Dx, diagnosis; M, male; F, female; FAB, French American British, ELN = European Leukemia Net; sHAM = sequential high dose cytarabine and mitoxantone; TAD = Thioguanin/Ara-C/Daunorubicin; HD = healthy donor;

**Figure 5:**
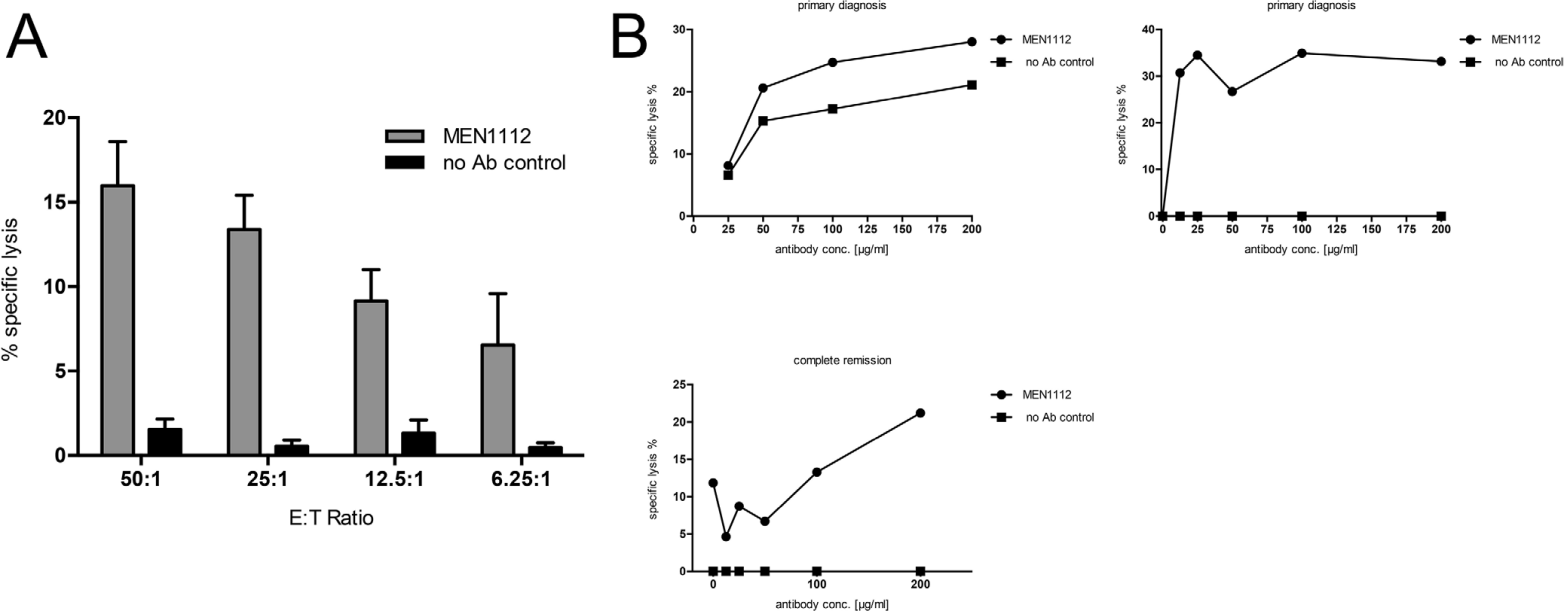
MEN1112 is able to mediate lysis of primary AML cells (**A**–**B**) Standard 4-8h-Cr^51^ release cytotoxicity experiments using primary AML cells: (A) Coculture of primary AML cells with HD NK cells and MEN1112 (10 μg/ml) at different E:T ratios (50:1-6.25:1). Data represent mean ± SEM of 5 experiments. (B) Coculture of primary AML cells with either autologous PBMCs from primary diagnosis (PBMC:AML cell ratio 80:1; upper left panel) or autologous NK cells from either primary diagnosis or complete remission (NK:AML cell ratio 20:1; upper right and lower left panel). MEN1112 was titrated in a 4-step serial dilution starting from 200 μg/ml. Target cells and NK cells without antibody served as negative control.

PETHEMA = Idarubicin + All *trans* retinoic acid (ATRA); ^†^ % specific lysis at highest MEN1112 concentration; n.d., not determined; n.a., not applicable; PT 1–7 are depicted in Figure 4; PT 8-12 are depicted in Figure 5A; PT13-15 are depicted in Figure 5B; the last column refers to MEN1112 lysis against target cells in the specific experimental set-ups.

### MEN1112 induces lysis of primary AML cells in an allogeneic and autologous setting

Cytotoxicity of MEN1112 was tested against primary AML cells in an allogeneic and in an autologous system. First, HD NK cells were coincubated with primary AML cells and MEN1112 in 4 h Cr^51^ release experiments. MEN1112 induced cytotoxicity against primary AML cells in an E:T ratio-dependent manner (*n* = 5, Figure 5A and Table 2). However, cytotoxicity was lower compared to experiments using AML cell lines (see Figure 2), which might be explained by a higher spontaneous background lysis and potential evolving resistance mechanisms of primary AML cells. In an attempt to mimic the *in vivo* situation, we finally tested MEN1112 in an autologous set-up, using NK cells and primary AML cells from the same donor. First, we used CD33/CD34-depleted PBMCs from primary diagnosis as effector cells and corresponding CD33^+^/CD34^+^ cells as target cells. In this setting, only marginal lysis was detected (Figure 5B upper left panel and Table 2). In further experiments isolated NK cells from either primary diagnosis or from time of complete remission were used as effector cells. Indeed, we observed MEN1112-mediated cytotoxicity in this setting (Figure 5B upper right and lower left panel and Table 2). These results closely resemble the heterogeneous picture obtained with NK cells from AML patients in coculture with AML cell lines (see Figure 4).

## DISCUSSION

Antibody-based immunotherapeutic strategies are currently entering the clinic for treatment of hematological malignancies including AML. Until now, CD33 was the most prominent and most often targeted antigen in AML [1, 2]. However, its rapid internalization into the cell upon receptor cross-linking makes it an unsuitable target for conventional antibody formats. Therefore, slow- or non-internalizing target antigens are needed to efficiently induce Fc-mediated effector functions. In the current study, we are the first to evaluate CD157 as a novel target antigen in AML. We could show that CD157 is expressed in 97% of AML patient samples with comparable prevalence and expression intensity at relapse compared to primary diagnosis. This is important, as numerous antibody-based immunotherapeutic strategies are preferentially used in relapsed/refractory patients. Furthermore, persistent expression of CD157 from primary diagnosis to relapse would provide the option for targeted therapy at any stage of the disease.

In our analysis correlating target antigen expression with genetic and molecular disease characteristics, we found equal expression intensities throughout all genetic risk profiles, with a tendency for higher expression in the ELN adverse group where novel immunotherapeutic approaches are most urgently needed. This makes CD157 an attractive target for immunotherapy in AML. Another important aspect for targeted immunotherapy is the expression of CD157 on LICs, which are supposed to be the major source of relapse. Although CD34^+^/CD38^−^ LICs showed significantly lower expression of CD157 compared to AML bulk cells, the expression level was within the range of the AML cell lines used, indicating that this level is sufficient for recognition. Moreover, for CD33-targeted immunotherapy it was shown *in vitro* that target antigen expression levels only impair lysis kinetics but do not influence overall response to therapy [19, 20], suggesting that LICs can be eliminated through CD157-targeted therapies. As shown by us and others, CD157 is expressed on CD34^+^ progenitor cells of the healthy BM compartment [17, 18]. The recently initiated phase I trial in relapsed/refractory AML (NCT02353143) will address the question of dose and wanted on-target cytotoxicity versus unwanted off-tumor hematotoxicity.

NK cells are promising effector cells for antibody-mediated target cell lysis. Through the binding of their Fc part to Fc receptors on NK cells, antibodies of the IgG1 and IgG3 class can induce ADCC [21]. However, the physiological affinity between the Fc receptor of the NK cell and the Fc part of the antibody is low, to avoid unspecific and overwhelming immune reactions [22]. In recent years, a lot of effort has been made in engineering monoclonal antibodies to increase the affinity of the Fc part to the Fc receptor to induce stronger ADCC responses. Indeed, we could show that MEN1112 induced higher lysis of U937 target cells compared to its parental fucosylated analogue. Compared to Rituximab-mediated lysis against CD20-expressing RAJI cells, MEN1112 triggered more efficient lysis of OCI-AML3 target cells, despite equal antigen expression and especially at low E:T ratios. Obinutuzumab, a glycoengineered anti-CD20 antibody, has already demonstrated to induce stronger effector functions and target cell lysis compared to Rituximab [23, 24]. Moreover, MEN1112 was able to mediate cytotoxicity of primary AML cells in an allogeneic setting. This is of major importance, as it has been shown that AML cells are able to suppress immune responses through different mechanisms like secretion of immunosuppressive factors (i.e. arginase) [25]. Using NK cells from AML patients, MEN1112 induced ADCC in AML cell lines in 33% (3/9) samples tested. In an autologous setting using NK cells from primary diagnosis in two cases and from complete remission in another case, MED1112 induced ADCC responses were heterogeneous. Our data suggest that NK cells in AML are potentially impaired and are unable to mediate ADCC. During the course of the disease, NK cells might regain some of their function, albeit to a highly variable degree. In combinatorial studies using Rituximab in combination with lenalidomide, an increase of NK cell function including CD16 upregulation was observed [26]. We hypothesize that combinatorial strategies using NK cell-recruiting antibodies with immunostimulatory substances might increase treatment success. Nevertheless the development strategy might be better clarified upon the availability of first-in-human (FIH) trial results.

In summary, we could validate CD157 as a commonly expressed target antigen in AML and AML LICs at time of primary diagnosis and relapse. MEN1112 induced efficient ADCC responses against CD157-expressing AML cell lines and primary AML cells. Effector functions were increased compared to its fucosylated parental analogue. However, we could confirm previously reported NK-cell attenuation in AML. The relevance of our *in vitro* findings has to be addressed in a FIH trial. A phase 1 study has recently been initiated evaluating the use of MEN1112 in relapsed/refractory AML patients (NCT02353143).

## MATERIALS AND METHODS

### Patients, diagnostics and AML risk stratification

After written informed consent in accordance with the Declaration of Helsinki and approval by the Institutional Review Board of the Ludwig-Maximilians-Universität (Munich, Germany), peripheral blood (PB) or BM samples were collected from HDs and patients with AML at primary diagnosis or relapse. Patient characteristics are summarized in Table 2. Mononuclear cells from AML patients and HDs were isolated by density gradient centrifugation (Biochrom, Berlin, Germany) and either cryoconserved at < −80°C in 90% FCS and 10% dimethyl sulfoxide (Serva Electrophoresis, Heidelberg, Germany) or immediately used.

At diagnosis, a standard analysis of all samples was performed centrally at the Laboratory for Leukemia Diagnostics, University of Munich. This included cytomorphology, cytogenetics, fluorescence *in situ* hybridization and molecular genetics, performed as described previously [27–29].

Combined cytogenetic and molecular risk stratification groups were assigned in accordance with the European LeukemiaNet (ELN) guidelines [30].

### Surface expression analysis

Surface expression of CD157 was assessed by flow cytometry (Navios, Beckman Coulter, Krefeld, Germany) using a fluorochrome-conjugated monoclonal antibody (SY11B5; e-Bioscience, San Diego, California). CD157 expression on CD34^+^/CD38^−^ cells from BM of AML patients and HDs was analyzed using the following antibodies: CD45 (J33), CD34 (581), CD38 (LS198.4.3) and CD33 (D3HL60.251). Corresponding isotype controls were used. All antibodies were purchased from Beckman Coulter (Krefeld, Germany). A CD45^DIM^/SSC^LOW^ gate was used to limit the analysis to myeloid progenitor cells.

Surface expression analysis of CD157 on AML cell lines and CD20 (clone 2H7, BioLegend, San Diego, California) on RAJI cells was performed on a BD LSR II (Becton Dickinson, Heidelberg, Germany). Staining was performed according to the manufacturer's instructions.

MFI values were determined using FlowJo (Version 9.4.11) (Tree Star Inc., Ashland, Oregon), and surface expression intensity (MFI ratio) was determined as previously described [20].

### Cell lines

All AML cell lines and the lymphoma cell line RAJI were purchased from DSMZ (Deutsche Sammlung von Mikroorganismen und Zellkulturen; Braunschweig, Germany) and cultured in RPMI1640 (PAN Biotech, Aidenbach, Germany), supplemented with 10% FCS and 1% penicillin/streptomycin/glutamine (GIBCO life technologies, Darmstadt, Germany), at 37°C and 5% CO_2_. Cell lines were screened by short tandem repeat (STR) profiling at DSMZ (Braunschweig, Germany) and tested for mycoplasma contamination at regular intervals.

### MEN1112

The parental MEN1112 antibody was generated using Phage Display at Alere TM (San Diego, California). Binding of the lead candidate to cynomolgus and human Bst1/CD157 was confirmed by flow cytometry. Humanization of MEN1112 mouse Fab variable heavy (VH) and variable light (VL) amino acid sequences was carried out by JN Biosciences of Mountain View, California with proprietary sequence analysis and modeling methods. The humanized variable domain sequences were cloned into the Lonza GS expression construct with human heavy (IgG1) and light (kappa) constant region amino acid sequences to generate a stable cell line. The MEN1112_19C4 antibody producing cell line was generated using the Lonza GS System transfected into the BioWa Potelligent cell line by electroporation. Clones were selected according to the Lonza GS selection process. Positive clones were adapted to serum free conditions and clone 19C4 was selected, characterized and adapted for production. The stable cell line produced humanized non-fucosylated antibody against CD157. MEN1112 antibody-producing cells were grown in 1-litre culture and supernatant was quantified for production and then purified on protein A column. Purified antibody was tested for quantity and quality by spectrophotometry (Nanodrop) and sodium dodecyl sulfate polyacrylamide gel electrophoresis (SDS-PAGE), respectively.

### Antibody-dependent cellular cytotoxicity (ADCC)

MEN1112-mediated cytotoxicity was determined in standard 4–8h Cr^51^ release assays. Target cells (AML cell lines or primary AML cells) were labeled with 50 μCi Cr^51^ (Hartmann Analytic, Braunschweig, Germany) for 90 minutes at 37°C. NK cells from HDs or AML patients at primary diagnosis or complete remission (CR) were negatively isolated (NK cell isolation kit, Miltenyi Biotech, Bergisch Gladbach, Germany) and added to the target cells at different E:T ratios (50:1–3.125:1) in triplicates. For autologous experiments, effector cells were either negatively isolated NK cells or CD33/CD34-depleted PBMCs. MEN1112 or Rituximab were added at a final concentration of 10 μg/ml. Rituximab was used as a non Fc-engineered control in some experiments as it represents a clinically established, therapeutic antibody with a validated target. Spontaneous lysis (Cr^51^-labeled target cells without NK cells and antibody) and maximum lysis (Cr^51^-labeled target cells directly on measurement plate) were determined in sextuplicates.

Specific lysis was calculated using the following formula:

$$\text{specificlysis}=\frac{\text{experimental cpm}-\text{spontaneous cpm}}{\text{maximal cpm}-\text{spontaneous cpm}}*100$$

After 4–8 hours of incubation at 37°C, supernatants were transferred to the measurement plate (Lumaplate, Perkin Elmer, Waltham, Massachusetts), dried overnight and analyzed using a TOPCount (Perkin Elmer, Waltham, Massachusetts) reader. For the determination of EC_50_ values, NK cells were added at an E:T ratio of 25:1, and MEN1112 was titrated in a 12-step serial dilution (1:4) starting from 300 μg/ml. Target cells and NK cells without antibody served as background control.

### Flow cytometry

Analysis of MEN1112 cytotoxicity against healthy cells was performed in either of the following experimental set ups: A) MEN1112 or an irrelevant Fc-engineered control antibody were added to PBMCs at different concentrations (1.7–0.007 μg/ml) and incubated for 20–24 hours. B) CD34^+^ BM cells (CD34 Microbeads, Miltenyi Biotech, Bergisch Gladbach, Germany) from a HD or positive-selected CD14^+^ monocytes (CD14 Microbeads, Miltenyi Biotech, Bergisch Gladbach, Germany) were incubated with negative-selected NK cells from an independent donor at an E:T ratio of 3:1 for 24 hours. MEN1112 or an irrelevant Fc-engineered control antibody were added at 5 μg/ml. (A+B) Target cells and NK cells without antibody served as background control. Cultures were harvested, stained with fluorochrome–labeled antibodies against CD33 (clone WM53) and CD56 (clone HCD56, BioLegend, San Diego, California) or CD16 (clone 3G8, BioLegend, San Diego, California) and analyzed by flow cytometry (BD LSR II; Becton Dickinson, Heidelberg, Germany). The percentage of CD33^+^ cells in untreated cultures relative to MEN1112 or the irrelevant Fc-engineered control antibody treated cultures was used to determine the percentage of lysis.

### Statistical analysis

The significance of differences between the two halves of CD157 expression was determined by Pearson's chi-squared test. The significance of differences between unpaired samples was determined by the two-tailed Mann-Whitney U test. For pairwise comparison, the two-tailed Wilcoxon signed rank test was used. Dose response curve fits were performed using the GraphPad Prism software version 4 (GraphPad Software, Inc., La Jolla, California). Calculations were performed in either GraphPad Prism or IBM SPSS Statistics for Windows (Version 21.0, Armonk, New York). Results are shown as means ± standard error of mean (SEM) or as indicated. Statistical significance was considered for *p* < 0.05.

## SUPPLEMENTARY MATERIALS FIGURES



## Abbreviations

AML: acute myeloid leukemia
NK: natural killer; antibody dependent cellular cytotoxicity
PBMC: peripheral blood mononuclear cell; bone marrow
PB: peripheral blood
MFI: median fluorescence intensity
LIC: leukemia initiating cell
E:T: effector to target
