# Supplementary material for: Targeting CD157 in AML using a novel, Fc-engineered antibody construct
Source: Oncotarget. 2017 Mar 9;8(22):35707–17. doi: 10.18632/oncotarget.16060 (PMC5482610; doi:10.18632/oncotarget.16060)
Supplement: Supplementary file 1 [file oncotarget-08-35707-s001.pdf]

Targeting CD157 in AML using a novel, Fc-engineered antibody construct

Supplementary Materials

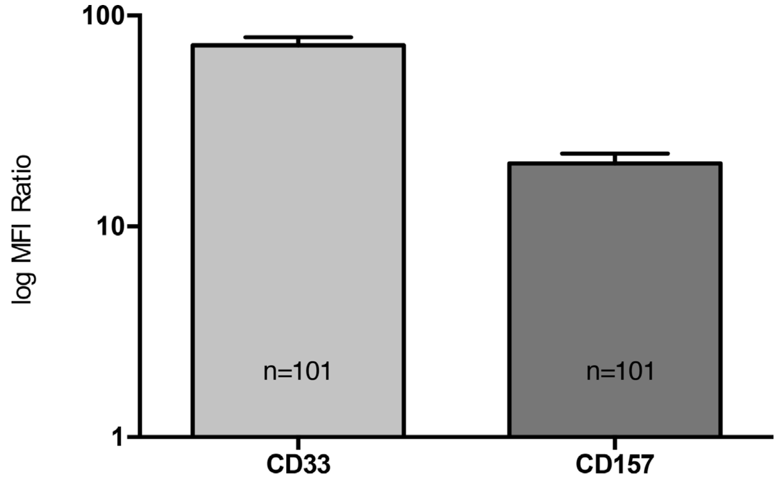

Supplementary Figure 1: CD157 expression intensity (MFI ratio) compared to CD33 in 101 primary AML patient samples.

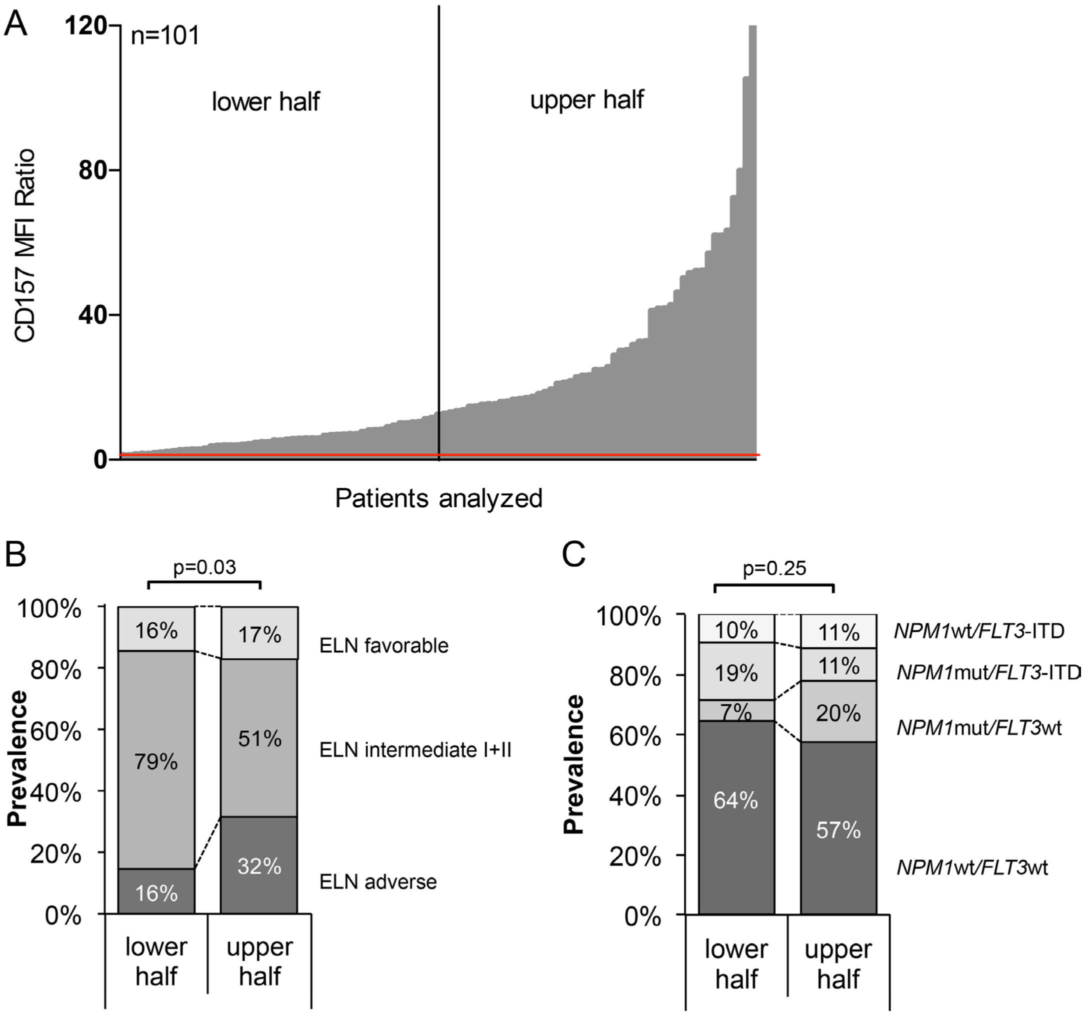

Supplementary Figure 2: Correlation of CD157 expression to molecular and cytogenetic characteristics in AML. (**A**) The patient cohort was divided into two halves based on expression intensity for correlation to (**B**) genetic risk groups according to the European Leukemia Net (ELN) classification and (**C**) to mutations in the *NPM1* and *FLT3* gene.
